# Factors influencing nurse practitioner panel size in team-based primary care: a qualitative case study

**DOI:** 10.1186/s12875-024-02547-6

**Published:** 2024-08-14

**Authors:** Ruth Martin-Misener, Faith Donald, Jennifer Rayner, Nancy Carter, Kelley Kilpatrick, Erin Ziegler, Ivy Bourgeault, Denise Bryant-Lukosius

**Affiliations:** 1 School of Nursing, Dalhousie University, 5869 University Ave, Box 15000, Halifax, NS B3H 4R2 Canada; 2 Daphne Cockwell School of Nursing, Toronto Metropolitan University (formerly Ryerson University), Toronto, ON Canada; 3 Alliance for Heathier Communities, Toronto, ON Canada; 4 Department of Family and Community Medicine, Health Policy, University of Toronto, Toronto, ON Canada; 5 School of Nursing, McMaster University, Hamilton, ON Canada; 6 Ingram School of Nursing, McGill University, Montreal, QC Canada; 7 School of Sociological and Anthropological Studies, University of Ottawa, Ottawa, ON Canada

**Keywords:** Primary care, Nurse practitioner, Panel size, Case study, Social determinants

## Abstract

**Background:**

Lack of access to health care is a worldwide public health crisis. In primary care it has led to increases in the implementation of nurse practitioners and heightened interest in their patient panel capacity. The aim of this study was to examine factors influencing nurse practitioner patient panel size in team-based primary care in Ontario, Canada.

**Methods:**

We used a multiple case study design. Eight team-based primary care practices including rural and urban settings were purposively selected as cases. Each case had two or more nurse practitioners with a minimum of two years experience in the primary care setting. Interviews were conducted in-person, audio recorded, transcribed and analysed using content analysis.

**Results:**

Forty participants, including 19 nurse practitioners, 16 administrators (inclusive of executives, managers, and receptionists), and 5 physicians were interviewed. Patient, provider, organizational, and system factors influenced nurse practitioner patient panel size. There were eight sub-factors: complexity of patients’ health and social needs; holistic nursing model of care; nurse practitioner experience and confidence; composition and functioning of the multidisciplinary team; clerical and administrative supports, and nurse practitioner activities and expectations. All participants found it difficult to identify the panel size of nurse practitioners, calling it— “a grey area.” Establishing and maintaining a longitudinal relationship that responded holistically to patients’ needs was fundamental to how nurse practitioners provided care. Social factors such as gender, poverty, mental health concerns, historical trauma, marginalisation and literacy contributed to the complexity of patients’ needs. Participants indicated NPs tried to address all of a patient’s concerns at each visit.

**Conclusions:**

Nurse practitioners have a holistic approach that incorporates attention to the social determinants of health as well as acute and chronic comorbidities. This approach compels them to try to address all of the needs a patient is experiencing at each visit and reduces their panel size. Multidisciplinary teams have an opportunity to be deliberate when structuring their services across providers to meet more of the health and social needs of empanelled patients. This could enable increases in nurse practitioner panel size.

**Supplementary Information:**

The online version contains supplementary material available at 10.1186/s12875-024-02547-6.

## Background

Access to health care is a worldwide public health crisis. The supply and productivity of the primary care workforce, including nurse practitioners, (NPs) is critical to its resolution [1–3]. Panel size is a metric which refers to the number of patients receiving care from a single provider in one year [4]. Though commonly assessed in physician practices [5–8], its use in NP practice is less frequent but increasing [9]. Panel size and composition is also of interest in the context of primary care teams and is regarded as a key component of high-performing teams [10–12].

NPs are advanced practice nurses with graduate education that includes clinical training to assess, diagnose and manage patients with acute and or chronic illness [3]. NPs function as the most responsible provider for their own patient panels or provide care for patients in a collaborating physician’s panel [9]. Estimates of NP panel size range between 500 and 1000 patients [9]. These panel size numbers are less than what is typical for physician patient panels leading some to question the cost effectiveness of NPs [13].

Research has shown that the presence of an NP in a physician’s practice increases the physician’s patient panel size [4–6]. However few studies have investigated the nature of the patient panels of NPs or the factors that influence panel size and composition. Use of electronic databases, as well as validated disease burden scores, or first-voice perspectives in this research is sparse [9]. Since NPs are autonomous primary care providers in most of North America and many countries around the world [14], understanding the factors that influence their panel size is important for health workforce planning.

In Canada NPs are employed in all provinces and territories and their numbers are growing [15]. The province of Ontario was the first to formally introduce and regulate NPs 25 years ago and continues to have the largest number of NPs in the country [16]. It is also the province with the longest and most varied experience with various models of interprofessional team-based primary care [17–20].

### Aim

Our study aim was to explore the factors influencing NP patient panel size in team-based primary care in Ontario, Canada.

## Methods

### Study design

We used a multiple case study design to enable understanding of the phenomena of panel size in the real-life context of rural and urban primary care practices [21]. Cases were defined as team-based primary care practices that included two or more NPs with at least two years experience in the setting.

**Funding** The study was funded by the Ontario Ministry of Health and Long-term Care. The grant number was 06695.

### Recruitment of cases and participants

In consultation with an advisory panel of health care administrators and providers, we discussed the various team-based primary care models in Ontario and decided to include Aboriginal Health Access Centres, Community Health Centres, Family Health Teams and Nurse-Practitioner Led Clinics all of which had been in existence for at least 10 years [17–20]. The research team purposively selected eligible practices in rural and urban locations. To enable onsite data collection, practices were located within five hours driving from a large city.

An information letter explaining the study was emailed to the senior administrator in each potential case site. A follow-up telephone call confirmed the practice met eligibility criteria and obtained organizational permission to conduct the study, including the processes for participant recruitment. Each primary care setting and participant received a small honorarium.

### Data collection and analysis

Individual interviews with providers and staff explored participants’ perceptions of the barriers and facilitators to increasing NP patient panel size. Interviews were conducted in-person by a research team member between March and November 2015. A small number of interviews were conducted by telephone when requested by participants. Interviews were 30 to 50 min in length and included member checking by summarising key points [22]. To promote data saturation, data collection occurred one case at a time [21]. Interviews were conducted in person at each site over two-to-three-days, recorded and transcribed. The interview guide is available as a supplemental file.

Transcripts were coded deductively and inductively using content analysis [23]. NVivo 12 was used to manage the data [24]. First, two research team members independently coded four transcripts, compared coding, and discussed emerging themes. Remaining transcripts were independently coded by four team members. Emerging and evolving themes were discussed and refined at research team meetings, including one with the advisory panel.

When reporting the findings, the descriptor “most participants” was used for approximately 75% or more participants, “many participants” for approximately 50–74%, “some participants” for approximately 25–49% and “few participants” for less than 25%. This approach protects participant confidentiality and provides an indication of how many participants expressed a viewpoint without making interpretations beyond the sample [25].

## Results

Forty participants including 19 NPs, 16 administrators (inclusive of executives, managers, and receptionists), and 5 physicians were interviewed. Their characteristics are reported in Table 1. Many NPs (60%) had graduate education, most (72%) were employed fulltime, and, with one exception, had three or more years experience as an NP. Many self-identified as being female (56%) and their ages ranged from 39 to 46 years. NPs reported spending between 4 and 4.8 days per week providing direct patient care.

**Table 1:** Characteristics of primary care practice settings and participants

	Aboriginal Health Centre (*n* = 2)	Community Health Centre (*n* = 2)	Family Health Team (*n* = 2)	Nurse Practitioner-Led Clinic (*n* = 2)
**Practice Setting**				
Geography	1 rural, 1 urban	2 urban	1 rural, 1 urban	1 rural, 1 urban
Years in Operation	› 10 years	› 10 years	5–9 years	‹ 5 years
**Participants**				
Administrators	*n* = 5	*n* = 4	*n* = 4	*n* = 4
Physicians	*n* = 2	*n* = 1	*n* = 1	*n* = 1
Nurse Practitioners	*n* = 4	*n* = 5	*n* = 4	*n* = 5
Average age (years)	45	44	39	47
Education	Diploma *n* = 1 Master’s *n* = 2 Doctorate *n* = 0 Other *n* = 1 Missing = 0	Diploma *n* = 3 Master’s *n* = 1 Doctorate *n* = 0 Other *n* = 0 Missing = 1	Diploma *n* = 3 Master’s *n* = 1 Doctorate *n* = 0 Other *n* = 0 Missing = 0	Diploma *n* = 1 Master’s *n* = 4 Doctorate *n* = 0 Other *n* = 0 Missing = 0
Experience in primary care (years) primary care (years)	0–2, *n* = 0 3–5, *n* = 1 › 5, *n* = 3	0–2, *n* = 0 3–5, *n* = 1 › 5, *n* = 3	0–2, *n* = 0 3–5, *n* = 1 › 5, *n* = 2	0–2, *n* = 1 3–5, *n* = 3 › 5, *n* = 1

Factors influencing NP patient panel size were identified at patient, provider, organizational, and systems levels along with eight sub-factors (Table 2). Participants from Aboriginal Health Access Centres and NP-Led Clinics indicated NPs had their own patient panel whereas participants from Family Health Teams and Community Health Centres indicated NPs and physicians shared a patient panel.

All participants found it difficult to identify the number of patients in NPs’ panels commenting it was “a grey area.” Panel size estimates ranged from 300 to 1800 across the eight settings. Some participants assumed that expectations for NP panel sizes were externally determined, for example, by government or health service organizations. Others perceived panel size was determined at the primary care practice level and or by each provider individually.

All participants indicated that numbers alone could not capture the complexities of many of their patients’ lives, even when social factors were included as a diagnosis. As illustrated in the following quote, this, in combination with NPs’ deep commitment to patient-centred preventative care, inevitably meant longer appointment times and likely smaller panel sizes. These concepts are further considered in the following subfactor sections organized as Patient Factors, NP Factors, Organizational Factors and System Factors.

*We really try hard here to put the diagnosis in and even poverty comes up or social isolation*,* so all of those factors. But even those things don’t capture the complexities. So*,* I think determining a panel size based on that and a philosophy of patient-centred preventative care— rather than acute episodic all the time. That means longer appointment times*,* less— maybe less patients* [NP, Nurse Practitioner-Led Clinic].

**Table 2:** Factors and subfactors influencing nurse practitioner panel size

**Patient Factors** Complexity of Patients’ Health and Social Needs
**Nurse Practitioner Factors** Holistic Nursing Model of Care Nurse Practitioner Experience and Confidence
**Organizational Factors** Multidisciplinary Team Roles Clerical and Administrative Supports and Activities Expectations Regarding NP Activities
**Systems Factors** Restrictions on NP Scope of Practice NP Remuneration

### Patient factors influencing NP Panel Size

The composition of the patient population and the complexity of their needs was the only patient related factor influencing NP panel size.

#### Complexity of health and social needs

Across all cases, most interview participants emphasized that the number one factor limiting NP panel sizes was the complexity of the health and social needs of patient populations. As one Family Health Team administrator explained; “*The more complex your patients are*,* the more time you’re spending with them*,* the higher their needs and then consequently it becomes more difficult to manage a larger panel*.”

Many participants stressed that the length of appointment times was dependant on the needs of patients. Although the primary care receptionists knew most patients well and tried to predict the length of time needed for appointments, this was often not possible. The full extent of patients’ needs was often not disclosed or discovered until the time of the appointment with the NP. When this occurred, it required NPs to choose between staying on schedule or extending the length of the appointment in order to address the presenting patient’s needs. Participants indicated that NPs chose to capitalize on the opportunity to address the needs of the patient especially if the patient was unlikely or unable to return for regular follow up.

*“You’re really only ready to give that injection but now you have 10 other issues*,* and you want to make sure you get through them because this patient may or may not come back for their follow up and it’s so important. I think that can really impact how your whole day looks”.* [NP, Community Health Centre]

Participants recognized that health care funders wanted primary care practices to increase the size of NPs’ patient panels and the panels of the practice as a whole. However, participants indicated this was difficult to do without compromising their capacity to address the health and social needs of the patients already in NPs’ panels. As the following quote illustrates, they described this as the tension between providing quality care versus quantity. For participants, establishing and maintaining a supportive relationship with patients already in their practice was fundamental to providing quality care.

*“I don’t know how you could have a relationship or how you could support the patient if they have 7 to 10 minutes or whatever the number might be. I recognize that they have expectations of us*,* being the funding body*,* that they need data*,* they need numbers. It’s a struggle with providing quality care versus quantity*,* especially when our patients come in with early onset aging and a higher representation of chronic diseases at an early age and issues with housing*,* financial*,* poverty —all those things. We manage health care*,* but in addition to health care*,* support for all the other areas— and it takes time.”* [Administrator, Aboriginal Health Access Centre].

Participants identified that NPs provided primary care for patients with complex health and social needs. This included “patients *with four or more chronic diseases with multiple complaints on any one day.”* Most participants stressed that while multiple chronic illnesses were a component of the challenges patients were experiencing, mental health and substance use concerns and socioeconomic factors compounded the complexity of their lives. The following two quotes illustrate this.

*“We draw from a range of socio-economic status areas in (city) and that affects many things. We have a high incidence or prevalence of mental illness*,* and a lot of issues are complicated through the presence of their mental illnesses {…} or make management more difficult. I’d say that’s a challenge for all our practitioners. {…} You know*,* you get it all just sort of wrapped together; complexities due to older patients or patients with multiple meds*,* and then you wrap that up with socioeconomics and mental illness— it’s huge*.” [Administrator, Family Health Team]

*That just doesn’t mean the medical complexity*,* that also means the social complexity. If that client is substance-using*,* then I may give them a medication for an infection and they will go get that medication and then come back and say*,* you need to keep this medication*,* and then run away again*,* and I don’t know if they’ll come back or not. It’s totally the medical and behavioural complexity of that individual that certainly limits how many people I see in a day*.

[NP, Community Health Centre].

Participants further elaborated that gender, literacy, prior unattachment to a primary care provider, historical trauma and marginalisation also contributed to complexity and therefore the need for more support and more time.

*“With the NPs*,* the responsibilities that they see and manage every day with complex patients. We have complex patients probably at a higher number and with early onset aging and historical trauma. Our patients come in with a lot more — they need a lot more support*.” [Physician, Aboriginal Health Access Centre].

*“We did have new families*,* and even with those patients*,* it was chronic issues. They were unattached and marginalized*,* and they’d never seen any primary care provider for years. And they were the frequent flyers between hospital emergency and the walk-in clinics*.” [NP, NP-Led Clinic].

In summary, participants were united in their view that the complex health and social needs of patients in their primary care settings required longer appointment times with the NP, and this in turn reduced NPs’ panel size capacity.

### Provider factors influencing NP panel size

Two provider factors influenced NP panel size: NPs’ holistic nursing model of care and their experience and confidence.

#### Holistic nursing model of care

Half of NP and administrator participants in all four models of team-based primary care identified that the holistic nature of the care NPs provide was an influence on their patient panel size. A physician explained that NPs’ ability to look at patients holistically originated from nursing culture and is a value-add that they bring to primary care.

*“I think one of the great things NPs add to primary care*,* and it’s something that’s important to all of us*,* is something of value in nursing culture*,* which is to see people holistically as people who have needs and who come in with fears and anxieties. I think that’s a value that our whole health system needs more of.”* [Physician, Community Health Centre].

Providing holistic care was linked to scheduling appointment times that enabled NPs to take time to nurture a relationship built on trust and to address the multiple health and social concerns of patients. An NP explained it as follows:

*“If we can be patient-centred and take the time with people to address their issues*,* listen to them*,* you’re going to get more done. You spend a lot more money when a person ends up in crisis than doing prevention and really listening carefully to people and having that relationship with them. A lot of patients come here and the biggest thing they say they notice is that we spend the time—we listen. Their doctor may have had 10 minutes with them. So*,* they really appreciate that and they’re more apt to follow through on your suggestions because they feel you’ve listened to their problem and have some trust.”* [NP, NP-Led clinic].

#### NP Experience and confidence

Most participants indicated that experience in the role and in primary care practice influenced NP panel size. This is illustrated in the following quotes from NP and administrator participants.

*“When they first started*,* they had hour-long appointments with the clients because*,* first of all*,* they didn’t know the computer system*,* and their primary care skills weren’t as fresh.”* [NP, Community Health Centre].

*If an NP is more novice and really having to deal with a big pile of complexity with inter-related problems and symptoms—that really does affect panel size. It affects the amount of time that the NP takes and really having to lean on his or her colleague to get advice and support.”* [Administrator, Community Health Centre].

Development of confidence occurred over time, and through application of theoretical knowledge and exposure to clinical issues in the practice setting. Having opportunities to discuss questions and decisions with a colleague enabled confidence. When patients presented with complexities that required NPs to consult other providers, these consultations impacted the number of patient appointments.

The connections between experience, confidence, and an ability to practice at a faster speed are summarised by an administrator in the following quote.

*“What I have noticed is because of the comfort and confidence that a more experienced NP has*,* he or she will be able handle a situation around a prescription or diagnosis much more quickly*.” [Administrator, Community Health Centre].

### Organizational factors influencing NP panel size

There were three organisational factors influencing the panel size of NPs: multidisciplinary team roles; clerical and administrative supports, and expectations regarding NP activities.

#### Multidisciplinary team roles

Most NP and physician participants and many administrator participants identified that multidisciplinary teams enabled increases in NP panel size because patients could receive care from other team members. For example, dieticians provided nutrition counselling for patients with diabetes, pharmacists assisted patients with smoking cessation and social workers enabled patients to make connections with community resources and supports. Participants identified that it was important for the multidisciplinary team to think deliberately about each team member’s contribution to patient care. They indicated that when patient needs were matched with team members with the expertise to meet those needs, the panel size of NPs and the panel size of the entire practice could be increased.

If we involve more multidisciplinary team members, for instance, having a diabetic program in place or if you have a pharmacist, more registered practical nurses involved, and social work more involved, I can see NPs having more patients and expanding the panel size more.

[Administrator, NP-Led Clinic].

Participants offered several examples of health care providers, who, through their education and team roles, provided specific skills and services to meet needs of patient groups. They discussed the importance of enabling all team members to practice to their full scope of practice and making deliberate decisions about involving providers with specific competencies in the care of specific patient populations. The following quote illustrates this concept.

*“Truly using every provider to their full scope of practice and not booking tasks and activities that could go to another provider. So*,* when I think about our NPs*,* they’re a pretty hot commodity. We need them to see specific things with specific clients with specific issues and if they’re booking up their schedules with a blood pressure recheck or —name your episodic thing—it does take away time from being able to see other patients. So that full scope is really incredible.”* [Administrator, Community Health Centre].

Some participants identified the importance of providers reflecting on their own professional and personal scope of practice as well as the scope of practice of other team members when planning how to address the needs of patients in the practice. As one NP explained

*“I always think— what am I best able to provide the patient? I’m not a social worker*,* I’m not a great CBT (cognitive behavior therapy) counsellor— so why try to spend time counselling when I can send them to somebody else*,* find them resources.”* [NP, Family Health Team].

Further to this, some participants indicated that multidisciplinary teams wanted opportunities to design, implement and evaluate new approaches to team collaboration. Many team members indicated that collaboration improved team building and that it enabled problem-solving that led to operational changes that contributed to panel size growth.

*“What I’m seeing on our team*,* is really excellent team collaboration. If the team is really healthy*,* and functional*,* and can problem-solve and can do mutually supportive stuff—I think that’s a team that can handle a bigger panel size.” [Community Health Centre*,* Physician]*.

Participants noted that with resources, such as dieticians, social workers, registered practical nurses and pharmacists, some of the health teaching previously done by NPs was now being carried out by other team members. This provided time for NPs to have more patient appointments.

*“It evolves relative to practice size*,* the number of patients to be seen and to NP experience and confidence and availability of supports. An NP two years ago might have been doing smoking cessation on their own; now we have the support of the pharmacist to do that.” [Family Health Team*,* Administrator].*

#### Clerical and administrative supports and activities

Most participants indicated that clerical, and administrative staff were essential for enabling NPs to increase their panel size. Many identified that their practices had standard times for different types of appointments. For example, patients needing a complete physical examination or who had mental health concerns would be booked for a one-hour appointment. Most other appointments were booked for 30 min. Participants noted that most patients were well known by clerical staff, and they knew how to adjust appointment duration times according to what patients were anticipated to need. The following quote exemplifies this perspective.

*“I think each individual NP and the girls at the front have a background of all the patients— we know the patients well*,* and we know how to book them as far as time.” [NP -Led Clinic*,* Administrator]*.

Many participants indicated that administrative responsibilities were a component of NPs’ workload and ranged from 0.5 to 7.5 h weekly.

*“Of course*,* I have administrative time when I’m not seeing patients so during that time*,* I’m doing paperwork and reviewing labs and doing administrative things. If I had less administrative time*,* then maybe I would be able to see more clients but that’s part of the job”* [NP, Family Health Team].

For others, administrative work included activities such as program planning, leading meetings, quality improvement and community education/outreach activities. Some participants indicated that the time NPs spent on administration lessened the available clinical time.

#### Expectations regarding NP activities

Most participants indicated appointment-based, direct care within the primary care practice setting was how NPs spent most of their time. In some practices NPs were expected to offer specific types of appointments that impacted their panel size, for example, same and next day patient appointments. In some practices NPs were responsible to do activities requiring more time, for example, completing the telephone follow-up calls or routine pelvic examinations for the patients of all providers in the practice.

Many participants indicated NPs did house calls or street calls noting these required more time because travel was entailed. For example, an Aboriginal Health Access Centre administrator said, “*I think we book 1 ½ hours*.” The frequency of house-calls was on an as-needed basis, with one NP-Led Clinic NP reporting 28 in a year. Most NPs reported they did not have on-call responsibilities except for some who were on-call but only for critical lab results.

In summary, the three subfactors at the organizational level were composition and functioning of multidisciplinary healthcare provider teams, clerical and administrative supports and expectations regarding NP activities.

### Systems Level factors influencing NP panel size

The systems level factors that influenced the panel size of NPs were restrictions on NP scope of practice and NP remuneration.

#### Restrictions on NP scope of practice

At the time of data collection, systems level factors that restricted scope of practice included barriers to referring patients to specialists, restrictions on ordering some diagnostic tests, and the inability to prescribe some medications. As one Family Health Team NP explained having these restrictions removed would save time because “having *to ask can I order this or that— it just slows down the time*.” The removal of the requirement for a physician’s co-signature on specialist referral requests had an impact on increasing panel size as illustrated in the following quote. “*Being able to refer now without a physician’s co-signing— that opened up a position for me in a satellite office in (location) to help with patients who didn’t have a family physician*.

#### NP remuneration

Funding was also an important enabling factor identified by many NPs and physicians. One NP from a Community Health Centre summarized it as follows:

If we think of wages as a system thing and we think about how it impacts on recruitment and retention, you can’t grow your clinic if you don’t keep your staff. {…} So, if you support recruitment and retention with good wages, you can grow your panel size. But your NPs get a better offer, and they go off to a better paying organization, […], then you can’t grow primary care, you can’t grow your clinic.

## Discussion

Through this analysis we found that the most significant factor influencing NP panel size was the time needed to address the multiple complex health and social needs experienced by patients in their practice. To accommodate for this, NPs had appointment times that varied between 15 and 30 min and some that were booked for an hour or more. Participants identified that providing care for patients impacted by the social determinants of health was important. All participants supported the value of the principle of time allocation for NPs to address patients’ broader social needs—especially patients experiencing trauma, marginalization and high-risk environments. They emphasized that NPs were ideally suited to provide care for this patient population because of NPs’ holistic, comprehensive nursing approach that included taking the time to listen and fulsomely address patients’ concerns at each appointment. Participants emphasized that concerns about panel size should not compromise the comprehensiveness of the care NPs provide to patients with complex needs because it was important from a social justice standpoint, and it reduced the risk of adverse patient outcomes.

The association between the length of appointment time and complexity has been documented from patient [26] and provider [27–29] viewpoints. Patients impacted by social factors, such as poverty, are also disproportionately affected by medical comorbidities [30]. When discussing complexity, participants spoke briefly about patients’ multimorbidity and gave detailed accounts of the social determinants impacting patients for whom NPs provided care. It is possible that their focus on these factors was a confirmation bias of their knowledge of the nursing profession’s focus on social determinants of health. Similar findings from studies using patient profile [28, 30] and task allocation studies [31] make this explanation unlikely. NPs in this study prioritized identifying and taking action to address the social needs of their patients regardless of the time it took or impact it had on panel size. Although not a concern of participants in our study, the cost effectiveness of NPs’ longer appointment times has been criticized [12]. However, recent studies indicate NP-provided primary care for patients with complex health needs results in savings in downstream costs associated with hospital use [32, 33]. Primary care is the point in health care systems where these interventions should be provided - not in the emergency room or in hospital.

Participants in our study were mindful of the need to attend to NP panel size. However, they tended to regard panel size determination as an accountability required of funders, not a tool to improve how care delivery could be better organized in the practice [34]. They were cognizant of the challenges patients faced when accessing primary care, for example, transportation and childcare, and recognized the visit was a time-limited window of opportunity to address concerns. Therefore, participants prioritized giving patients the time required to have most or all of their needs met in a single appointment. This approach aligns with Levesque et al.’s [35] broad perspective on accessibility as an opportunity to identify healthcare needs and have those needs for services fulfilled. It is also consistent with the call to action of Browne and Tarlier, who said: “With even a small investment of time, NPs operating from a critical social justice perspective could for example, attend to the biomedical needs of patients and *at the same time* work towards countering or challenging the social or economic policies that affect biomedical issues” [36, p. 90]. Participants in our study prioritized their time for these patients but did not discuss policy work they were doing related to the social determinants of health.

While participants valued and made efforts to optimize the roles of multidisciplinary team members including registered and practical nurses, for the most part they did not discuss how the team could take a more deliberate approach to maximizing optimal functioning of all team members’ roles and the impact this could have on NP panel size. Such planning requires data, and we did not specifically ask participants to what extent they could mine electronic medical record data to inform this planning. Research has shown that NPs use EMR data to plan their own clinics [37], however, few practices are using this data to inform financial and organizational planning for the services a primary care clinic could offer and the roles of team members [38]. This could enable improvements in accessibility and equity.

### Limitations

Our study offers an in-depth description of the factors affecting NP patient panels in primary care in one Canadian province. Interview data were collected in 2015 before the COVID-19 pandemic and the increased use of virtual care in primary care. Future research should evaluate strategies for aligning team composition with patient population health and social needs as well as the potential impact of virtual care provision on NP panel size. Our study did not include patient interviews and not having their perspectives is a limitation. Detailed information about the structures and processes of these practice models other than what is presented in Table 1 was not obtained in our study and is a limitation to understanding the impact of context on panel size.

## Conclusion

Multiple factors influence NP patient panel size, particularly the complexity of the health and social needs of patients. NPs have a patient-centred, holistic approach that aims to address the social determinants of health for each patient when they access primary care. Panel size concerns should not compromise the comprehensiveness and equity of the care NPs provide. Better utilization of practice-based data may enable multidisciplinary teams to optimize panel size without compromising equity and quality of care.

### Electronic supplementary material

Below is the link to the electronic supplementary material.

Supplementary Material 1

## Data Availability

The qualitative data that support the findings of this study are not openly available. Participants did not consent to have their data shared.
