# Supplementary material for: Factors influencing nurse practitioner panel size in team-based primary care: a qualitative case study
Source: BMC Prim Care. 2024 Aug 14;25:304. doi: 10.1186/s12875-024-02547-6 (PMC11323452; doi:10.1186/s12875-024-02547-6)
Supplement: Supplementary file 1 — Supplementary Material 1 [file 12875_2024_2547_MOESM1_ESM.docx]

**Supplemental File**

**Questions for Interviews with Primary Care Administrators, Clinicians and Staff**

**Setting the Stage**

**►Introduce the topic area to be explored: e.g., “Today we’re going to talk about factors that influence patient panel size for nurse practitioners in this setting. Please think about the number and types of patients that the NPs care for, as well as the other activities that the NP carries out. The interview will be approximately 30 minutes in length. Thank you for participating in this study."**

**Confidentiality Reminder**

**► “To ensure confidentiality, please use invented names if you refer to specific examples that include a patient, family member, or staff. The information that you share during this interview is confidential. Your individual answers will not be identified and shared with your employer, colleagues, or patients. Please keep your contributions to the study confidential.”**

**Permission to audio record**

**► “I would now like to ask your permission to turn on the audio recorder.** The interviews will be audio recorded to ensure accuracy of data collected and transcribed with any identifying data removed. If you do not wish to be audio recorded, please tell me and I will take notes instead of the audio recording.**”**

**________________________________________________________________**

1. Think back to when the role of the NP was first introduced into this practice. At that time, what did you expect that the NPs would do?

***Probes:***

- 1. What did you think the NPs would be doing for patient care?
  2. What did you think the NPs would do other than direct patient care in the clinic? e.g., administrative, clerical, program planning and evaluation, long-term care/home visits

1. What types of patients did the NPs see when the role was first introduced? Has this changed?

***Probes*:**

a. all ages or specific ages

b. male, female, transgender

b. health status - e.g., acute or chronic; wellness/minor/more complex/multimorbidity

c. socio-economic status

1. What are the current areas of focus and expectations of the NPs in this practice?

***Probes***:

a. Please estimate the time that the NPs spend in these areas/activities

1. As the NPs gained more experience, did the activities of the NPs change? How did these changes influence the patient panel size for NPs?

***Probes:***

- 1. Why do you think this might be?
  2. Changes in clinic structure
  3. Full-time vs part-time
  4. Role clarity
  5. Continuity of care
  6. Most responsible provider for individual patients

1. Have NPs' activity changes influenced how you work with the NP and the supports that you provide?

***Probes***:

a. Level of autonomy of the NPs?

1. How have organizational factors influenced the patient panel size for NPs?

***Probes***:

a. Clinic infrastructure - e.g., number of exam rooms, location of and sufficient equipment, hours of day for patient care

b. Support personnel - e.g., scheduling appointments; escorting patients to the exam room; faxing; retrieving faxes; phlebotomy; on-site testing (urine, serum glucose)

c. Rostering/registration system - do the NPs have their patient panels or is the patient panel shared with others? How does that system affect NP patient panel size?

1. Types and roles of team members?

***Probes***:

a. What roles do the NPs play in the team?

b. How do these roles and activities influence NP patient panel size?

1. How does the wider system influence the patient panel size for NPs?

***Probes*:**

a. Legislation regarding the NP role

b. NP regulated scope of practice

c. NP funding

d. NP education

e. Population health

**9.**  In your opinion, what has the greatest impact on the number of patients that NPs see per day?

**10.**  In your opinion, what has the greatest impact on the number of patients that could be registered/rostered to NPs in your practice?

**11**. Is there anything else that you wish to add regarding factors that influence NP patient panel size?

**12**. What recommendations would you make to optimize NP patient panel size?

**13.** What do you think is the **single most important patient factor** that influences patient panel size?

**14.** What do you think is the **single most important NP factor** that influences patient panel size?

**15.** What do you think is the **single most important organizational factor** that influences patient panel size?

**Summary**

**► At the conclusion of the interview the interviewer will summarize the discussion points made by the participant and clarify points, if needed. The interviewer will also give the participant the opportunity to verify and clarify points and/or their perceptions.**

**The interview will finish with a thank you to the individual for participating and an appreciation of the contributions that the participant has made to better understanding NP patient panel size.**
